# Survival-Weighted Health Profiles in Patients Treated for Advanced Oral Cavity Squamous Cell Carcinoma

**DOI:** 10.3389/fonc.2021.754412

**Published:** 2021-09-29

**Authors:** Yao-Te Tsai, Wen-Cheng Chen, Cheng-Ming Hsu, Ming-Shao Tsai, Geng-He Chang, Yi-Chan Lee, Ethan I. Huang, Chiung-Cheng Fang, Chia-Hsuan Lai

**Affiliations:** ^1^ Department of Otorhinolaryngology-Head and Neck Surgery, Chang Gung Memorial Hospital, Chiayi, Taiwan; ^2^ Department of Radiation Oncology, Chang Gung Memorial Hospital, Chiayi, Taiwan; ^3^ Department of Otorhinolaryngology-Head and Neck Surgery, Chang Gung Memorial Hospital, Keelung, Taiwan

**Keywords:** oral cavity squamous cell carcinoma, quality of life, survival-weighted psychometric scores, life expectancy, quality-adjusted life expectancy

## Abstract

**Objectives:**

For patients with oral cavity squamous cell carcinoma (OSCC), particularly for those with advanced disease, quality of life (QoL) is a key outcome measure. Therefore, we estimated survival-weighted psychometric scores (SWPS), life expectancy (LE), and quality-adjusted LE (QALE) in patients with advanced OSCC.

**Methods and Materials:**

For estimation of survival function, we enrolled 2313 patients with advanced OSCC diagnosed between January 1, 2007, and December 31, 2013. The patients were followed until death or December 31, 2014. To acquire the QoL data, data from 194 patients were collected by employing the Taiwan Chinese versions of the Quality of Life Questionnaire Core 30 and Quality of Life Questionnaire Head and Neck 35 developed by the European Organisation for Research and Treatment of Cancer and the EQ-5D-3L between October 1, 2013, and December 31, 2017. The LE of the patients with OSCC were estimated through linear extrapolation of a logit-transformed curve. SWPS and QALE were determined by integrating the LE and corresponding QoL outcomes.

**Results:**

For the patients with advanced OSCC, the estimated LE and QALE were 8.7 years and 7.7 quality-adjusted life years (QALYs), respectively. The loss of LE and QALE was 19.0 years and 20.0 QALYs, respectively. The estimated lifetime impairments of swallowing, speech, cognitive functioning, physical functioning, social functioning, and emotional functioning were 8.3, 6.5, 6.5, 6.1, 5.7, and 5.4 years, respectively. The estimated lifetime problems regarding mouth opening, teeth, social eating, and social contact were 6.6, 6.1, 7.5, and 6.1 years, respectively. The duration of feeding tube dependency was estimated to be 1.6 years.

**Conclusions:**

Patients with advanced OSCC had an estimated LE of 8.7 years and QALE of 7.7 QALYs. SWPS provided useful information regarding how advanced OSCC affects the subjective assessment of QoL. Our study results may serve as a reference for the allocation of cancer treatment resources.

## Introduction

Oral squamous cell carcinoma (OSCC) is the sixth most common cancer in the world, and its incidence has been increasing, with an annual incidence approaching 500,000 (1, 2). In Taiwan, because of the high prevalence of betel nut chewing and cigarette smoking (3), OSCC is the fourth most common cancer among men. Approximately 60% of patients with OSCC present with locoregionally advanced disease (stage III or IV) at diagnosis (1), and the 5-year survival rate is only 10%–40% (2). Both OSCC and its treatments can significantly impair patients’ quality of life (QoL) and functional status. Conventionally, the outcome assessments for OSCC consider both physician and patient perspectives, with physicians objectively reporting survival, local control, and complication rates and patients subjectively reporting physical, emotional, social, and psychological outcomes (3). Patient reported outcomes are increasingly studied (4), and OSCC patients have reported varying degrees of physical problems [e.g., eating and speaking changes (5)], mental stress [e.g., fatigue, anxiety, and depression (6, 7)], and altered interpersonal relationships [e.g. social isolation, work impairment, and disrupted social relationships (5, 8)]. Among head and neck cancer (HNC) patients, OSCC patients experience the worst QoL and function (9). Patients with advanced OSCC frequently experience moderate to severe QoL and functional impairments attributable to their extensive tumor invasion or multidisciplinary treatments, such as ablative surgery and radiation therapy (9, 10). Surgery plus adjuvant radiotherapy can result in more severe and prolonged QoL disturbance compared with radiotherapy alone (11), and psychosocial and functional impairment may persist for a long time (5, 8). Hence, periodic review of QoL and the use of questionnaires may facilitate communication between patients and physicians and thereby optimize cancer treatments and nutritional interventions, potentially improving survival in patients with OSCC (12).

The quality-adjusted life-expectancy (QALE) that considers both survival and QoL is widely applied for cancer patient care and clinical research (13, 14). Studies have compared and quantified QALE in patients with HNC by estimating life expectancy (LE) and quality-adjusted LE (QALE) (14, 15). However, these studies enrolled highly heterogeneous samples and did not consider survival-weighted psychometric scores (SWPS). In the present study, we investigated the feasibility of estimating QALE and SWPS by combining mean QoL scores at various intervals with survival function in patients with advanced OSCC.

## Materials And Methods

### Patients

**Figure 1:**
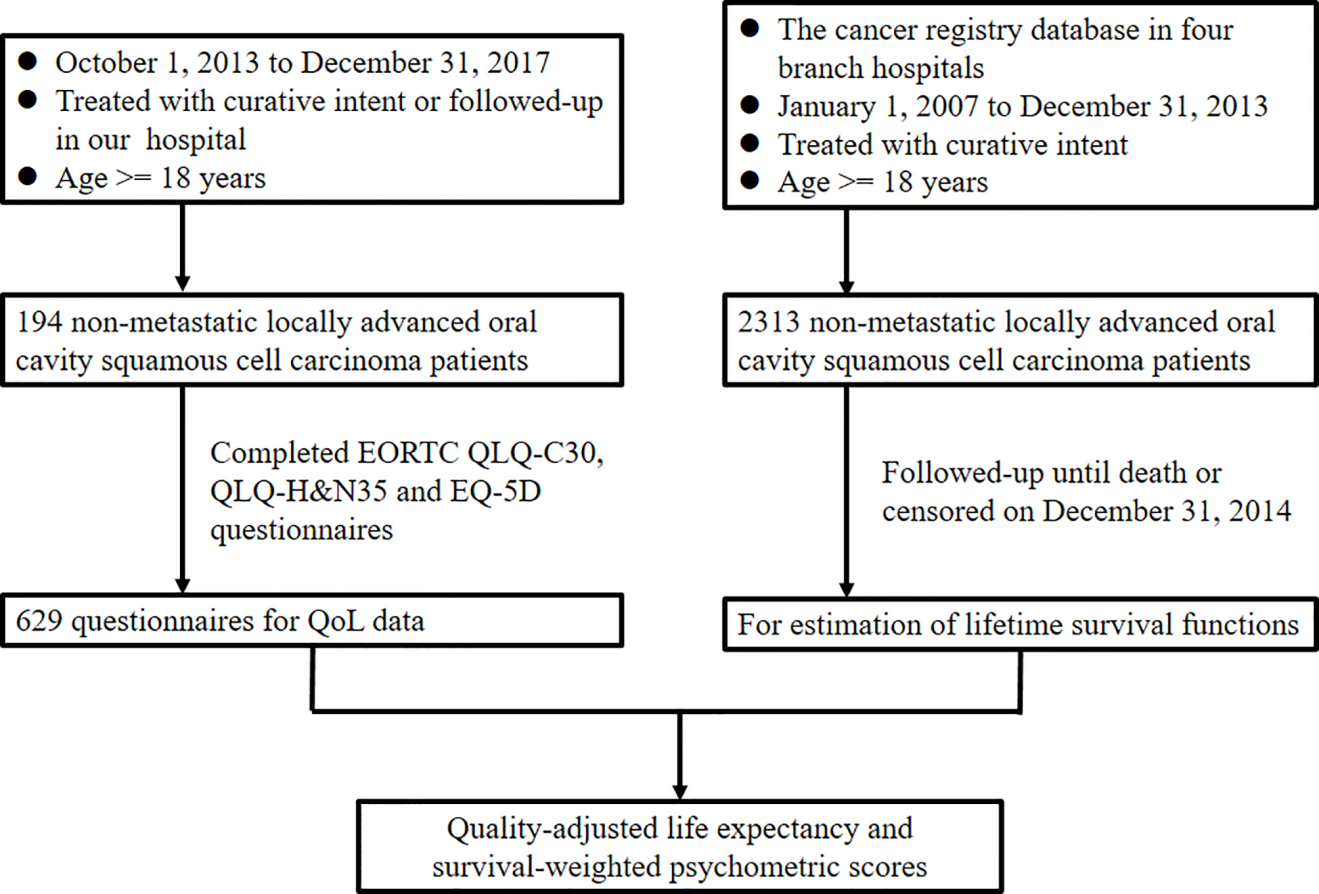
Study design flowchart. EORTC, European Organisation for Research and Treatment of Cancer; H&N, head and neck; QLQ, quality of life questionnaire; QoL, quality of life.

**Figure 1** presents the study flowchart. Patients diagnosed as having OSCC between January 1, 2007, and December 31, 2013, were retrospectively analyzed for survival estimation. Eligibility criteria were the following: (1) aged 18 to 75 years; (2) had newly diagnosed locally advanced OSCC (stage III or IV); (3) underwent curative treatments; and (4) had an Eastern Cooperative Oncology Group performance status of 0 to 2. We excluded patients who (1) underwent palliative treatment; (2) had a history of any cancer; (3) whose OSCC had already metastasized at diagnosis; or (4) had another cancer in addition to OSCC. Finally, a cohort of 2313 patients with advanced OSCC diagnosed during the study period was enrolled from our cancer registry database. Patients with OSCC who underwent treatments and follow-up at our hospital from October 1, 2013, to December 31, 2017, were prospectively enrolled for QoL questionnaire completion. Informed consent was obtained from all participants, and the institutional review board of our hospital approved the study protocol (No. 102-2668B). This study was performed in compliance with the tenets of the Declaration of Helsinki.

### Treatment Protocol

Each patient underwent a routine workup consisting of comprehensive history taking, physical examination, flexible fiberoptic laryngoscopy, plain chest radiography, abdominal sonography, and pretreatment computed tomography or magnetic resonance imaging of the head and neck. In addition, the computed tomography of chest will be arranged if there is any abnormal finding on the plain chest radiography, and the positron emission tomography/computed tomography scan will be performed in patients with stage IV disease or if there is any finding suspicious for metastasis in the aforementioned studies. All patients underwent either primary ablative surgery with adjuvant therapy or radiotherapy (RT)/chemoradiotherapy (CRT) with curative intent. The cancer staging manual of the American Joint Committee on Cancer (2010) was used for OSCC staging. The types of adjuvant therapy were determined by the tumor board conference according to institutional guidelines. The detailed adjuvant treatment guidelines in our institute and their comparison with the National Comprehensive Cancer Network guidelines have been reported by Lin et al. (16). In brief, patients diagnosed as having a pathologic T4 disease and single metastatic neck lymphadenopathy are provided adjuvant RT, whereas those diagnosed as having extranodal extension, multiple metastatic lymphadenopathies, or positive surgical margins are administered adjuvant CRT. If indicated, the intensity-modulated RT (2 Gy/d, 5 d/week) was used to treat patients, and the radiation dose was 60–66 Gy in an adjuvant setting and 70–72 in a definitive setting. Platinum-based agents were used if chemotherapy was indicated. All patients were regularly followed up, at which time questionnaires were completed. Follow-up visits occurred during years 1–3 every three months and in years 4+ every six months. At every follow-up visit, all patients were reviewed by speech-language pathologists and dietician and underwent complete physical examination including the fiberoptic laryngoscopy. Moreover, during the follow-up period, we executed head and neck magnetic resonance imaging or computed tomography at 6-month intervals during the first 2 years and annually thereafter.

### QoL Instruments

The Taiwan Chineseversions of the European Organisation for Research and Treatment of Cancer (EORTC) Quality of Life Questionnaire Core 30 (EORTC QLQ-C30) and EORTC Quality of Life Questionnaire Head and Neck 35 (EORTC QLQ-H&N35) were used to assess QoL (17–19); these instruments, in translation and after cross-cultural adaptation to a Mandarin-speaking population, have been validated (20, 21). Per the EORTC scoring manual, for both instruments, scores were linearly transformed; all scales (multiple or single item) were scored 0–100 (22). A higher functioning score and QoL scale score indicated high functioning or QoL. By contrast, high scores on the symptom scales indicated more severe symptoms.

The EQ-5D-3L; Taiwanese version) was employed to assess general health and analyze cost utility (23). The EQ-5D-3L has five domains (pain/discomfort, mobility, anxiety/depression, self-care, and activities of daily living) and three levels of classification (no, some, and extreme problems). The health information derived from the five domains was transformed into health-related utility values by the time trade-off method (24). The utility value indicated the degree of general health status on a scale from 0 to 1, with 0 representing death and 1 representing perfect health.

### Statistical Analysis

Numbers with percentages were used for categorical variables, and means with standard deviations were used to indicate continuous variables. The survival duration of the 2313 patients from the cancer registration database was defined as the duration from the date of curative treatment to death or censoring on December 31, 2014. We then plotted the Kaplan–Meier curves for overall survival estimation. On the basis of the life table of the general population in Taiwan, the Monte Carlo method was applied to determine the survival function of the reference population (matched for age and sex) (25). Linear extrapolation of a logit-transformed curve of the survival ratio between patients with OSCC and the reference population was performed to obtain the LE of the patients with OSCC (25–27). Kernel smoothing of the QoL data from 194 patients was applied to estimate average QoL function (27). The functional disabilities or symptoms were plotted against time at the beginning of curative treatment. From then until the attainment of every QoL follow-up data point, the survival outcomes were combined with the psychometric scores or utility values to calculate the SWPS or QALE (3). In brief, the utility values or psychometric scores at different time points were multiplied with the corresponding lifetime survival probabilities over the course of cancer to obtain the quality-adjusted survival curve, of which the area under the curve would be the QALE or SWPS (28). The LE implies the expected total duration of living under a certain degree of unhealthy status after the treatments; the QALE can be interpreted as the expected total duration of living under a perfect healthy condition after the treatments. Each SWPS in a psychometric item can be interpreted as the expected total duration of living under a condition with a problem in that item after the treatments. The utility value was assumed to be 1 for the reference population during the study period. Hwang et al. proposed a minimum sample size of 50 for generating the mean QoL function curve (27). Considering the 7-year follow-up data and extrapolation to 50 years of survival, we estimated the LE, QALE, and SWPS of patients with OSCC. SPSS Statistics for Windows, version 17.0 (SPSS Inc., Chicago, IL, USA) was used for statistical analysis, and p <.05 was considered to indicate statistical significance. Survival extrapolation was performed using iSQoL [http://sites.stat.sinica.edu/tw/isqol/; validated in (13, 29, 30)].

## Results

### Patient Characteristics

**Table 1** presents the patients’ baseline characteristics. The survival data of 2313 patients with OSCC were used for lifetime survival estimates. Another 194 patients were selected for QoL questionnaire completion. Among the enrolled patients, the most common stage of OSCC was stage IVA (n = 1413, 61.1%), followed by stage III (n = 602, 26.0%) and stage IVB (n = 298, 12.9%). Two thousand one hundred five (91%) patients received ablative surgery as their primary treatment modality; 1088 (47%) patients underwent postoperative adjuvant CRT, and 448 (19.4%) patients received adjuvant RT. Given the presence of the unresectable T4b disease, significant underlying comorbidities [e.g. end-stage liver disease (31) and severely reduced ejection fraction (32)**]**, and the patient’s willingness, approximately 10% of patients underwent definitive RT/CRT as their primary treatment (33, 34). **Table 2** presents the results of the 629 valid responses to the EORTC QLQ-C30 and QLQ-H&N35 completed by 194 patients with OSCC, which were stratified by time periods: post-treatment <1 year, 1−3 years, and >3 years.

**Table 1:** Baseline patient characteristics.

Variables	OSCC patients (n = 2313)	Patients completed QoL questionnaires (n = 194)
Age at diagnosis (years, mean ± SD)	51.9 ± 10.9	52.4 ± 9.8
Gender		
Male	2147 (92.8%)	192 (99.0%)
Female	166 (7.2%)	2 (1.0%)
Overall stage		
III	602 (26.0%)	56 (28.9%)
IVA	1413 (61.1%)	113 (58.2%)
IVB	298 (12.9%)	25 (12.9%)
T classification		
T1	124 (5.4%)	16 (8.2%)
T2	496 (21.4%)	46 (23.7%)
T3	428 (18.5%)	23 (11.9%)
T4A	988 (42.7%)	84 (43.3%)
T4B	277 (12.0%)	25 (12.9%)
N classification		
N0	870 (37.6%)	105 (54.1%)
N1	514 (22.2%)	28 (14.5%)
N2	918 (39.7%)	60 (30.9%)
N3	11 (0.5%)	1 (0.5%)
Curative treatment		
Surgery	2105 (91.0%)	174 (89.7%)
Adjuvant CRT	1088 (47.0%)	123 (63.4%)
Adjuvant RT	448 (19.4%)	51 (26.3%)
Curative RT/CRT	208 (9.0%)	20 (10.3%)

CRT, chemoradiotherapy; QoL, quality of life; OSCC, oral cavity squamous cell carcinoma; RT, radiotherapy; SD, standard deviation.

**Table 2:** The mean scores of the EORTC QOL scales in different periods of time.

	T1 scores (± SD)	T2 scores (± SD)	T3 scores (± SD)
EORTC QLQ-30			
Global quality of life	51 (± 22)	58 (± 20)	60 (± 22)
Physical functioning	73 (± 24)	80 (± 21)	87 (± 15)
Emotional functioning	72 (± 26)	78 (± 23)	79 (± 24)
Cognitive functioning	78 (± 24)	75 (± 20)	77 (± 19)
Social functioning	59 (± 35)	65 (± 29)	72 (± 32)
Role functioning	75 (± 34)	80 (± 31)	88 (± 23)
Fatigue	41 (± 27)	27 (± 26)	25 (± 23)
Nausea/vomiting	10 (± 19)	03 (± 13)	04 (± 11)
Pain	35 (± 31)	18 (± 22)	14 (± 20)
Dyspnea	15 (± 23)	15 (± 22)	15 (± 23)
Insomnia	35 (± 36)	24 (± 30)	26 (± 30)
Appetite loss	28 (± 32)	13 (± 22)	11 (± 19)
Constipation	17 (± 22)	17 (± 27)	12 (± 19)
Diarrhea	13 (± 23)	7 (± 15)	10 (± 17)
Financial problems	46 (± 39)	44 (± 35)	36 (± 36)
EORTC QLQ-H&N35			
Pain	29 (± 26)	18 (± 24)	12 (± 14)
Swallowing	45 (± 28)	38 (± 25)	44 (± 26)
Senses (taste/smell)	30 (± 29)	32 (± 33)	17 (± 27)
Speech	34 (± 29)	29 (± 28)	35 (± 30)
Social eating	48 (± 30)	42 (± 31)	47 (± 34)
Social contact	26 (± 27)	22 (± 25)	27 (± 28)
Sexuality	33 (± 34)	29 (± 32)	24 (± 28)
Teeth	33 (± 37)	42 (± 41)	44 (± 34)
Opening mouth	49 (± 35)	47 (± 36)	54 (± 40)
Dry mouth	48 (± 37)	53 (± 35)	48 (± 34)
Sticky saliva	50 (± 35)	34 (± 33)	33 (± 35)
Coughing	33 (± 30)	31 (± 23)	26 (± 25)
Feeling ill	45 (± 35)	26 (± 26)	21 (± 25)

T1, within the first year after treatment beginning; T2, post-treatment 1−3 years; T3, post-treatment 3 years and thereafter.

EORTC, European Organization for Research and Treatment of Cancer; QLQ, quality of life; H&N, head and neck; SD, standard deviation.

### Survival Outcome, LE, and QALE

**Figure 2:**
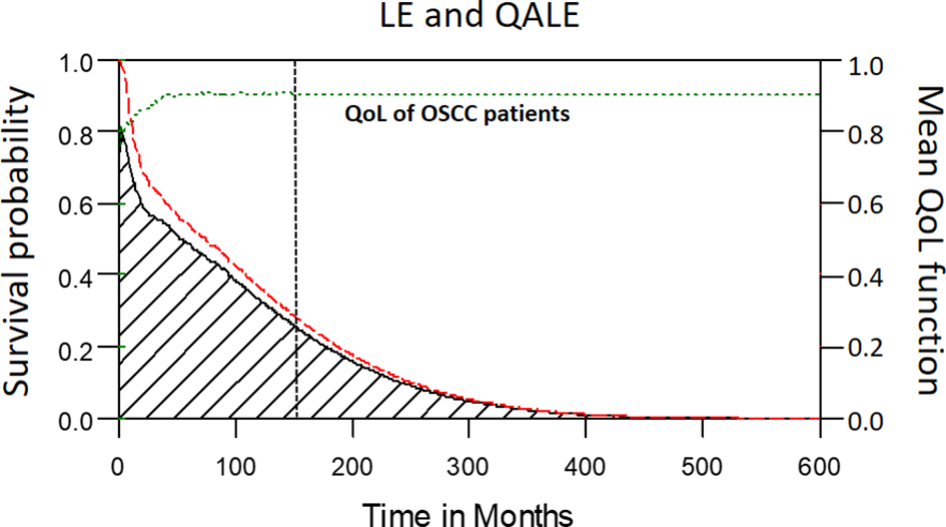
The mean QoL (utility) function (green dashed line) was multiplied with the corresponding lifetime survival probabilities (red dashed line) to obtain the quality-adjusted survival curve (black solid line). The area under the red dashed line is the LE. The area under the black solid line is the QALE. The vertical black dotted line stands for the starting month of extrapolation.

**Figure 3:**
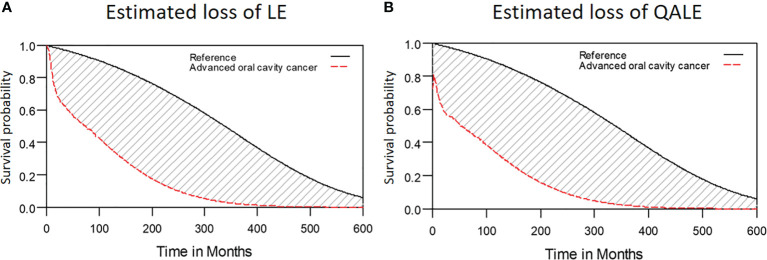
Estimated loss of LE and QALE for advanced OSCC patients. **(A)** Estimated loss of LE; **(B)** Estimated loss of QALE.

Among the 2313 patients with OSCC, the 5-year overall survival rate was 54.2% (median follow-up: 31.4 months; range: 0.7–97.1 months). The LE and QALE for the reference cohort in Taiwan is 27.7 years and 27.7 quality-adjusted life years (QALYs). In our cohort of patients with OSCC, the estimated LE and QALE was 8.7 years (95% confidence interval [CI]: 6.3–14.8 years) and 7.7 QALYs (95% CI: 5.5–13.1 QALYs), respectively (**Figure 2**); thus, the estimated loss was 19.0 years and 20.0 QALYs, respectively (**Figures 3A, B**, respectively).

### Symptoms and Impaired Function

**Figure 4:**
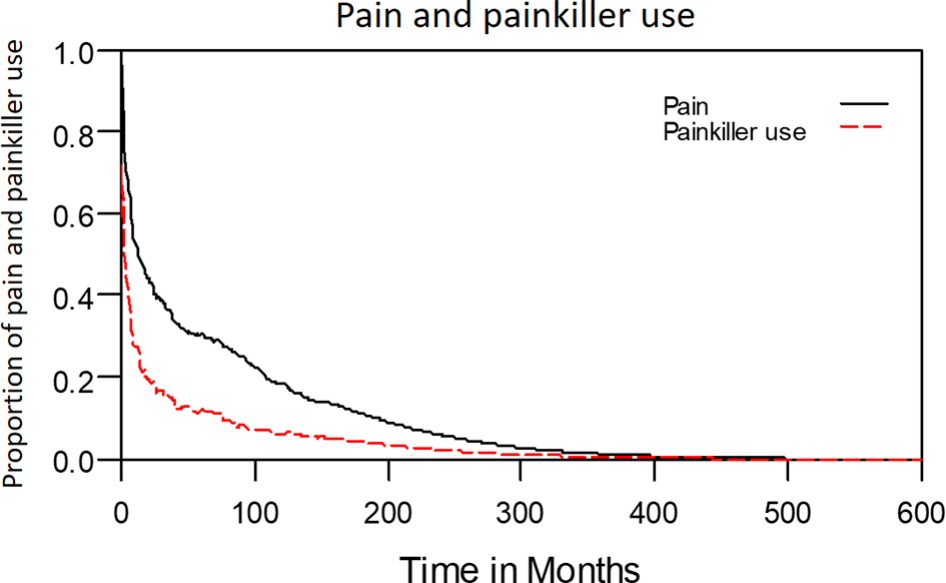
Dynamic changes in pain and painkiller use in patients with locally advanced OSCC.

**Figure 5:**
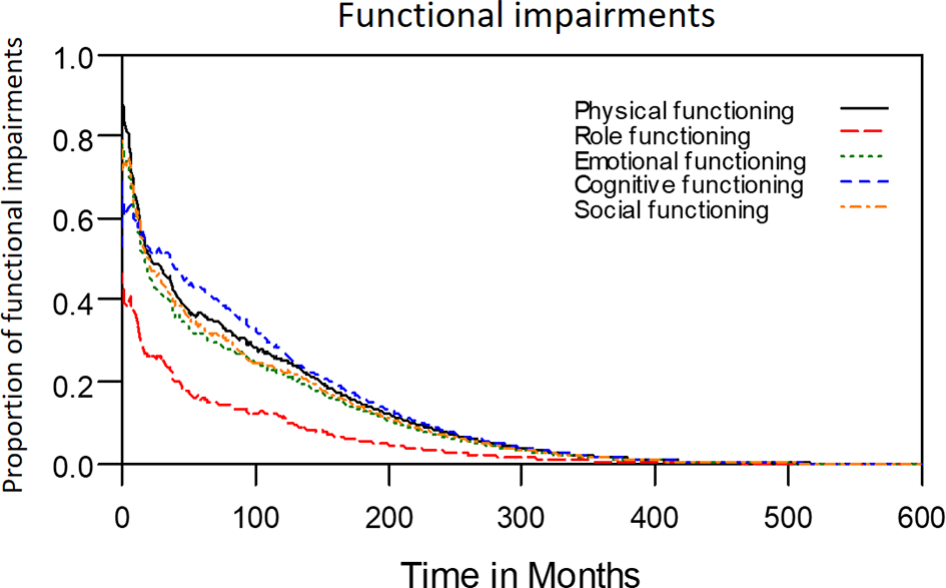
Functional impairments in patients with advanced OSCC. The estimated persistence of functional impairments is represented by the area under the quality-adjusted survival curve. Duration of functional impairments (years): Role—2.8; Physical—6.1; Emotional—5.4; Cognitive—6.5; Social—5.7.

**Figure 6:**
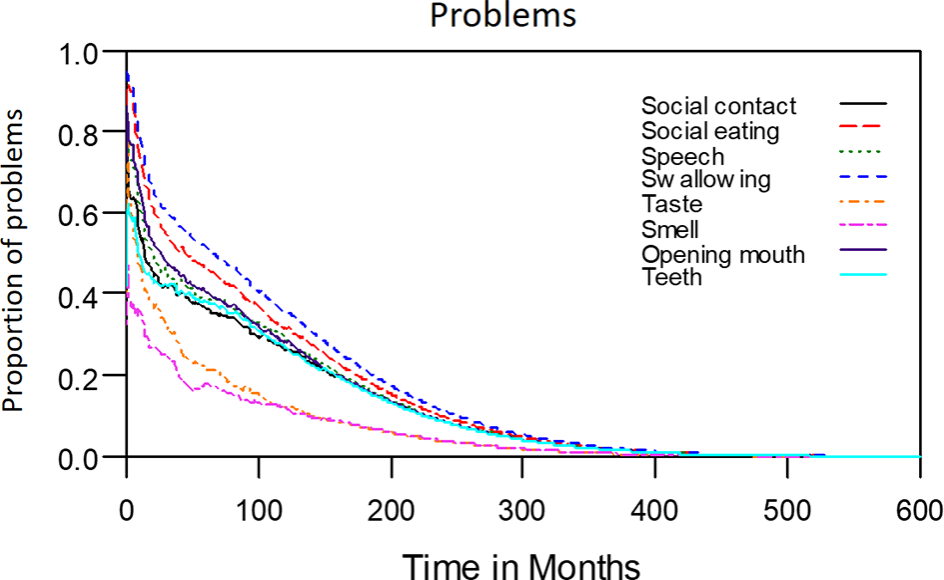
Problems in patients with advanced OSCC. The estimated persistence of impairments or problems are represented by the area under the quality-adjusted survival curve. Duration of functional impairments or problems (years): Taste—3.6; Smell—3.0; Speech—6.5; Swallow—8.3. Problem—years endured: Open mouth—6.6; Dentition—6.1; Social eating—7.5; Social contact—6.1.

**Figure 7:**
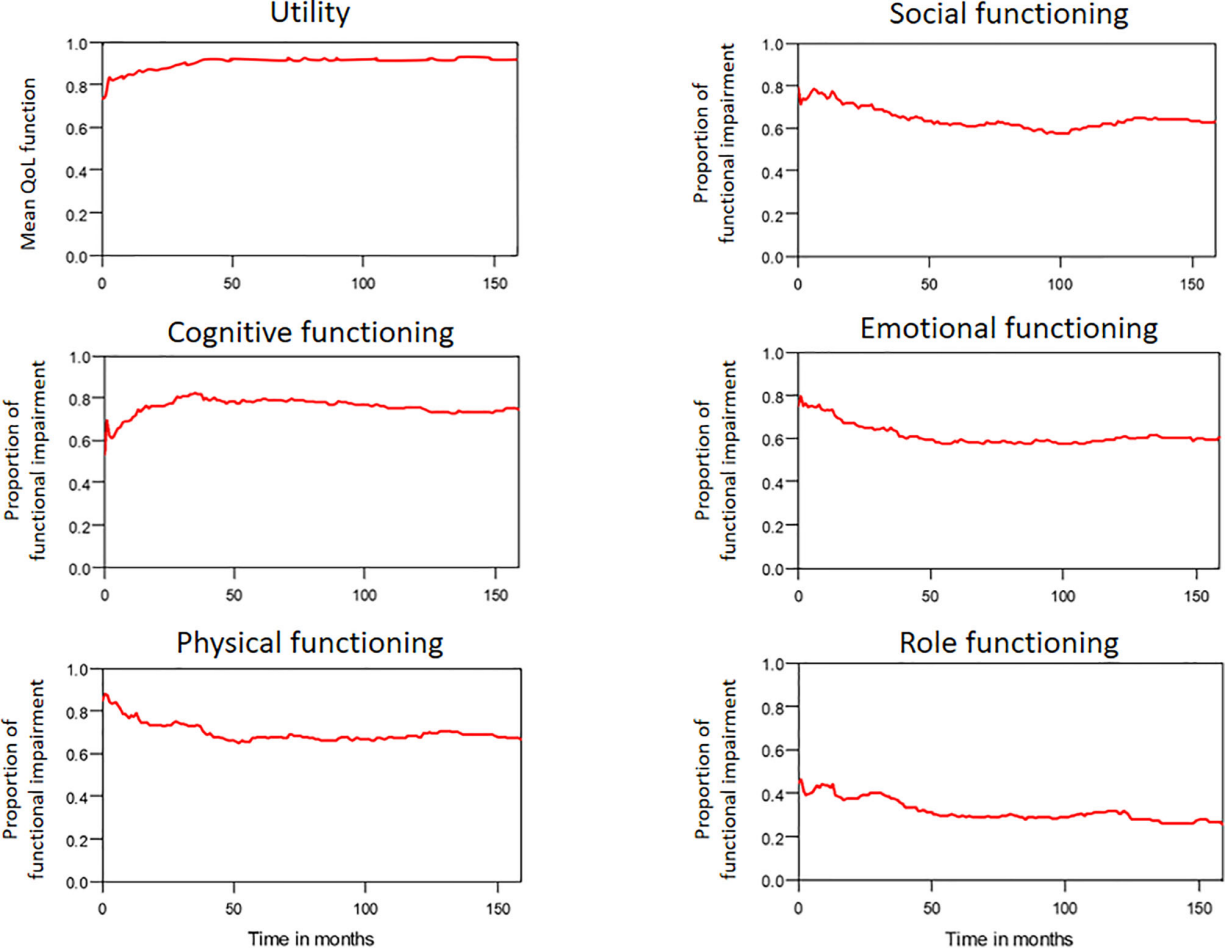
The trends of mean QoL (utility) function and functional impairments in patients with OSCC.

**Figure 8:**
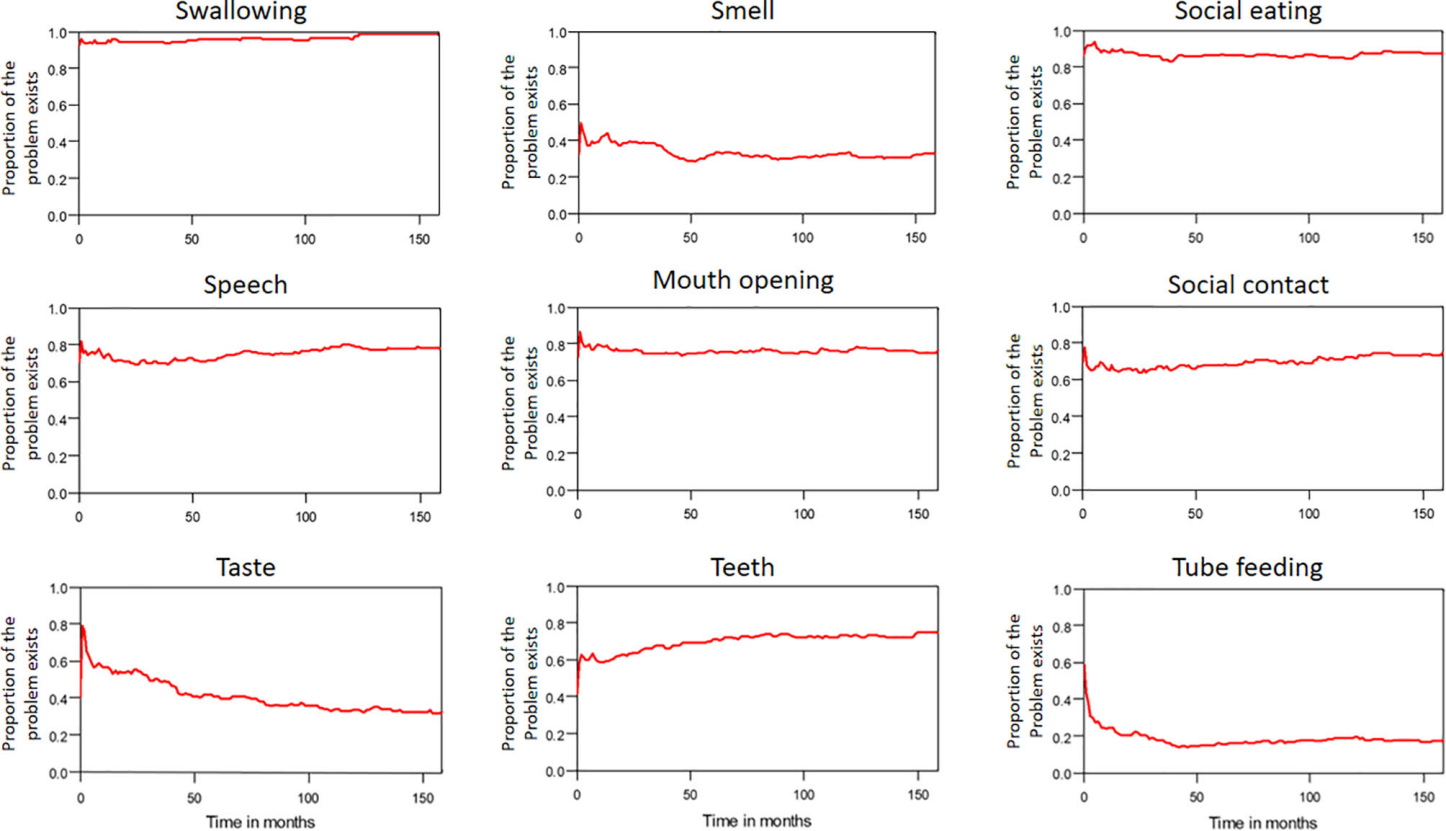
The trends of different problems in patients with OSCC.

The median period between curative treatments and questionnaire completion was 1.7 months (range: 1–158.2 months). We estimated that patients with OSCC experienced pain and consumed painkillers for 4.9 and 2.0 years, respectively (**Figure 4**). Regarding functional disabilities, the durations of impairments in cognitive, physical, social, emotional, and role functioning were estimated to be 6.5 (95% CI: 4.8-11.6), 6.1 (95% CI: 4.6-11.5), 5.7 (95% CI: 4.4-10.0), 5.4 (95% CI: 4.1-10.0), and 2.8 (95% CI: 2.0-4.9) years, respectively (**Figure 5**). The durations of impairments in swallowing, speech, taste, and smell were estimated to be 8.3 (95% CI: 6.4-15.0), 6.5 (95% CI: 4.9-11.7), 3.6 (95% CI: 2.7-6.6), and 3.0 (95% CI: 2.2-4.1) years, respectively (**Figure 6**). The patients experienced problems involving mouth opening, teeth, social eating, and social contact for an estimated 6.6 (95% CI: 5.0-11.4), 6.1 (95% CI: 4.5-11.0), 7.5 (95% CI: 5.7-13.3), and 6.1 (95% CI: 4.3-11.1) years, respectively (**Figure 6**). The estimated duration of tube feeding dependence was 1.6 (95% CI: 1.1-2.8) years. In addition, the dynamic changes of the utility values and functional impairments (**Figure 7**) as well as different problems (**Figure 8**) were also demonstrated.

### Extrapolation Validity

**Figure 9:**
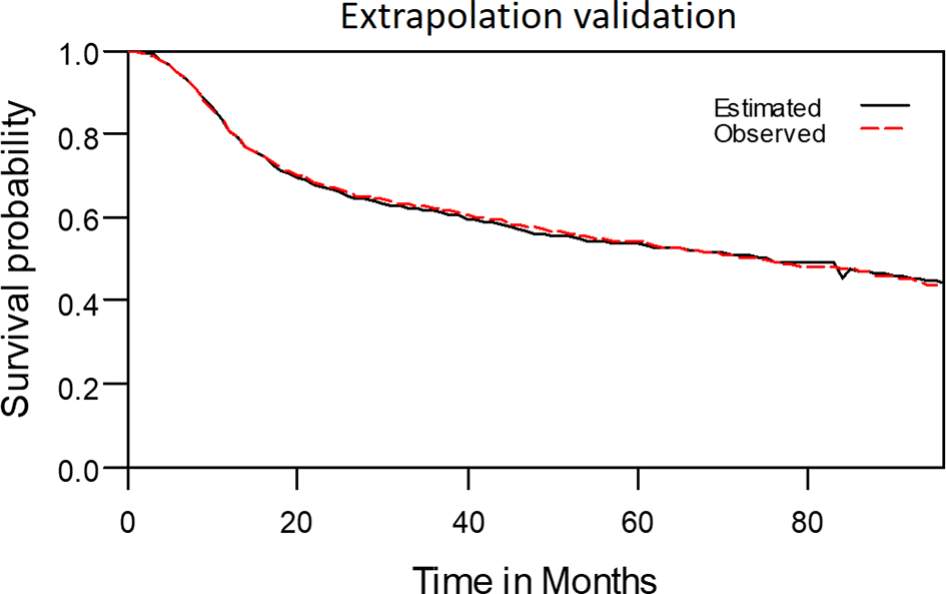
The observed 8-year survival curve and the estimated 8-year survival curve matched properly.

The model-extrapolated 8-year overall survival outcomes (using the initial 7-year follow-up data of 2313 patients) was compared with the survival outcomes measured using the Kaplan–Meier method. As shown in **Figure 9**, the observed survival data were highly consistent with the estimated survival curve. The mean ± standard deviation of estimated survival among the patients with OSCC was 58.9 ± 1.0 months, indicating a relative bias of only 0.3% from the observed survival (59.1 ± 0.8 months) at the end of the 8-year follow-up period.

## Discussion

Patients with locally advanced OSCC tend to have more symptoms, more severe functional disabilities, and greater reductions in QoL due to aggressive tumor extension and metastatic lymphadenopathy necessitating extensive surgical interventions or RT/CRT (4). Multidisciplinary management and advancements in treatment have facilitated the control of advanced OSCC (35); therefore, understanding the lifetime health burden of these patients is critical (36). To the best of our knowledge, this is the first study to describe QALE and lifetime symptoms or functional impairments in patients with locally advanced OSCC undergoing curative treatments. The estimated durations of problems concerning the teeth, mouth opening, social contact, and social eating all exceeded 6 years, consistent with results from a previous study (37). This may be explained by the significant lasting changes in oral structures, facial appearance, and social adaptation after curative treatment. We also observed that all the QoL domains excepting role function impairment (2.8 years), namely social, emotional, cognitive, and physical functioning, were adversely affected for over 5 years. This finding may be ascribable to the relatively young age of the patients at diagnosis, as well as to their active social participation and sufficient family support. The LE and QALE of the average patient with OSCC in Taiwan are approximately 12.2 years and 11.3 QALYs, respectively (15). Our estimates of LE and QALE, which were lower, are reasonable because we enrolled only patients with locally advanced disease. By integrating the QoL data from the QLQ-C30, QLQ-H&N35, and EQ-5D-3L questionnaires with the survival function, we generated a multidimensional health profile from the patient perspective, enabling an intuitive understanding of the changes of QoL in patients with advanced OSCC. Of note, OSCC and its treatments negatively impact patient QoL, particularly in those treated with ablative surgery (11). Extensive surgery considerably changes the facial appearance of patients with HNC, causing problems in social eating, swallowing, and speech and leading to social isolation and depression (38, 39). Given that 90% of our patients were treated with primary ablative surgery, their prolonged functional disabilities may be partially explained by the impacts of this extensive procedure. This highlights the necessity of developing psychosocial rehabilitation strategies for patients with advanced OSCC (40).

Our study results may extend the literature concerning QoL function in patients with locally advanced OSCC in several regards. First, because disease severity and treatment courses may influence various facets of QoL, a comprehensive assessment of psychometric scores for each QoL facet may yield a more holistic view. For instance, acute cancer- or treatment-related symptoms, such as pain and nausea or vomiting, may resolve gradually during follow-up. By contrast, patients with OSCC may experience prolonged physical distress and social functioning impairment that may or may not recover even after long-term disease remission is achieved (41). Multidimensional assessment may reflect the changes in QoL after the diagnosis of OSCC and may thus increase the feasibility of using QoL as an endpoint of treatment efficacy (42). Because the substantial change of the patient’s QoL usually happened within the first 2 months after curative treatments due to the surgical morbidity, treatment related toxicity and its recovery, and most symptom burden tended to be stable after 1 year (43). Hence, we collected the QoL data more frequently in the first 2 months after treatments for better estimation of QALE or SWPS. In addition, the QoL after the last data collection time point is assumed to be the same thereafter. Accordingly, even >50% of QoL data points came from within the first 2 months after treatments, it may cause little impact on the lifelong extrapolation. Second, patients’ subjective judgments of QoL may change over time (44). Hence, we used the extrapolation method, which entails a simulation approach, for estimating the lifetime survival function. By integrating the extrapolated survival outcomes and the psychometric data, we acquired the SWPS for lifetime QoL assessments in patients with advanced OSCC. Changes in QoL scores over time correspond to the cancer treatment courses and disease severity in patients with cancer, and the QoL profile is particularly informative regarding emotional distress, physical performance, and social function (45); these findings accord with ours. Overall, SWPS may constitute a comprehensive approach for determining the lifetime QoL function of patients with advanced OSCC.

The health costs and economic burden of OSCC are comparable with or higher than those of other cancers (46). Despite their poorer survival outcomes, the patients with advanced OSCC used more resources (corresponding to higher expenditures) than did those with early-stage OSCC. This is attributable to the need of this patient group for multidisciplinary treatment, supportive care, and palliative care following repeated relapse (47). The results of this study demonstrated that compared with the reference population, patients with locally advanced OSCC had substantial losses of LE (19.0 years) and QALE (20.0 QALYs). Given that the QALY metric is commonly used to assess value in health care decision-making (48), our data could yield useful information about resource allocation in advanced OSCC care.

This study has several limitations. First, the QALE and SWPS may have been overestimated for the following reasons. During extrapolation, the assumption of a constant level of QoL near the end of follow-up may have been distorted because real QoL usually declines with age (49). Moreover, patients who survived longer might have had a better QoL and completed more questionnaires (50). Further studies involving the administration of long-term QoL questionnaires and longer follow-up periods are warranted to confirm our findings. Second, the reference population utility was assumed to be 1 for the survival duration. Therefore, the loss of QALE among patients with OSCC may have been overestimated. Notably, Chung et al. indicated that women had an older mean age at diagnosis, less LE reduction, and a longer estimated QALE than men (15). However, our cohort had only two women with OSCC who completed the QoL questionnaires. Given that the computed tomography of chest was not routinely performed during staging workup in this study, small lung metastasis may have been underestimated and could negatively impact the patient’s prognosis and QoL (51). This potential confounding factor may need to be considered in the results interpretation. Another potential confounding factor is that the improvement of the surgeon’s technique and experience may lead to the better survival and QoL outcomes in patients who were treated in the later period of this study. Although our results involve intuitive assessment and appear reasonable, their interpretation should be made with these limitations in mind.

In conclusion, patients with advanced OSCC had an estimated LE and QALE of 8.7 years and 7.7 QALYs, respectively, and estimated LE and QALE losses of 19.0 years and 20.0 QALYs, respectively. The data on SWPS indicated that patients experienced multiple ongoing problems and functional disabilities over a long period of time following curative treatments. Future studies should evaluate whether information obtained from data on QALE and SWPS can be used to allocate health care resources and assess the impacts of surgery with different neoadjuvant or adjuvant protocols in patients with OSCC.

## Data Availability Statement

The raw data supporting the conclusions of this article will be made available by the authors, without undue reservation.

## Ethics Statement

The studies involving human participants were reviewed and approved by Institutional review board of Chang Gung Memorial Hospital (No. 102-2668B). The patients/participants provided their written informed consent to participate in this study.

## Author Contributions

C-HL and W-CC were involved in the conception and design. M-ST, C-MH, and G-HC were involved in the analysis and data interpretation. Y-TT and CHL drafted the manuscript. Y-CL, C-CF, and EH revised the manuscript critically for intellectual content. All authors contributed to the article and approved the submitted version.

## Funding

This study was supported by the Chang Gung Memorial Hospital (grant numbers: CORPG6D0251-3).The funding source had no role in the design of this study and had no role during its execution, analyses, interpretation of the data, or decision to submit results.

## Conflict of Interest

The authors declare that the research was conducted in the absence of any commercial or financial relationships that could be construed as a potential conflict of interest.

## Publisher’s Note

All claims expressed in this article are solely those of the authors and do not necessarily represent those of their affiliated organizations, or those of the publisher, the editors and the reviewers. Any product that may be evaluated in this article, or claim that may be made by its manufacturer, is not guaranteed or endorsed by the publisher.
